# Liposomes in Cancer Therapy: How Did We Start and Where Are We Now

**DOI:** 10.3390/ijms24076615

**Published:** 2023-04-01

**Authors:** Melody D. Fulton, Wided Najahi-Missaoui

**Affiliations:** 1 Department of Chemistry, College of Arts and Sciences, Washington State University, Pullman, WA 99164, USA; 2 Department of Pharmaceutical and Biomedical Sciences, College of Pharmacy, University of Georgia, Athens, GA 30602, USA

**Keywords:** liposomes, PEGylation, cancer, anticancer therapy, nanoparticle, targeted drug delivery

## Abstract

Since their first discovery in the 1960s by Alec Bangham, liposomes have been shown to be effective drug delivery systems for treating various cancers. Several liposome-based formulations received approval by the U.S. Food and Drug Administration (FDA) and European Medicines Agency (EMA), with many others in clinical trials. Liposomes have several advantages, including improved pharmacokinetic properties of the encapsulated drug, reduced systemic toxicity, extended circulation time, and targeted disposition in tumor sites due to the enhanced permeability and retention (EPR) mechanism. However, it is worth noting that despite their efficacy in treating various cancers, liposomes still have some potential toxicity and lack specific targeting and disposition. This explains, in part, why their translation into the clinic has progressed only incrementally, which poses the need for more research to focus on addressing such translational limitations. This review summarizes the main properties of liposomes, their current status in cancer therapy, and their limitations and challenges to achieving maximal therapeutic efficacy.

## 1. Introduction: Cancer and Chemotherapy

Cancer is the second leading cause of death and constitutes a major public health burden worldwide [1,2]. In the United States, it is estimated that one in three women and one in two men will be diagnosed with cancer in their lifetimes [3]. Prostate and breast cancers account for the most common non-cutaneous cancers among men and women, respectively, in addition to lung cancer [1]. In fact, the World Health Organization listed breast cancer as the most commonly diagnosed cancer worldwide in 2020 [4]. Cancer is classified according to its staging number, which helps clinicians (and patients) assess the extent of the tumor. The most clinically useful staging system is the TNM staging, which refers to tumor, node, and metastasis, respectively. This system was developed by the American Joint Committee on Cancer (AJCC) and has since been referred to as the AJCC TNM system [5]. The TNM staging system assesses the size of the tumor (T), the involvement of regional lymph nodes (N), and any evidence of distant metastasis (M). T is usually associated to a number that can be 0, 1, 2, 3, or 4. Numbers after the T (such as T1, T2, T3, and T4) describe the tumor size and/or cancer spread to nearby structures. A higher T number indicates a larger tumor size and/or increased cancer invasion to local tissues [5,6].

Treatment options for cancer depend on many factors, including the stage, age, and patient comorbidity. Prostatectomy and mastectomy are the first treatment options for more than 50% of men with prostate cancer and women with breast cancer. In addition to surgery, the treatment options usually include a combination of chemotherapy, radiation, and hormonal therapy for advanced stages of cancer [7]. Photodynamic therapy has emerged as a non-invasive and effective approach in the local treatment of cancer. A photosensitizer may be combined with a cancer drug. The activation of the photosensitizer by an appropriate wavelength of light results in the generation of reactive oxygen species that are capable of damaging and killing cancer cells. This process can also cause an immune response inside the tumor that can cause more cancer cells to die [8,9]. Although beneficial, surgery and radiation therapy are usually associated with debilitating side effects, such as incontinence and bowel complications in prostate cancer patients [10]. Female patients under hormonal treatment may suffer from menopausal-like symptoms, such as hot flashes, night sweats, osteoporosis, increased risks of cardiovascular diseases, and obesity [11].

The development of resistance to chemotherapy remains a major problem in the management of many cancer types [12]. The mechanisms of resistance to chemotherapy are still not completely understood, but include the activation of cellular survival pathways to inhibit cell death mechanisms, as well as possible epigenetic mechanisms that are yet to be fully elucidated [13]. Cancer cells may also exhibit multidrug resistance (MDR), which is most commonly associated with drug efflux mechanisms via ATP-binding cassette (ABC) membrane transporters. Examples of the most studied MDR transporters include P-glycoprotein (P-gp), which is overexpressed in various cancers and can bind and efflux a large number of anticancer drugs. Other examples include multidrug resistance-associated protein-1 (MRP1) and breast cancer resistant proteins (ABCG2), which are able to reduce the intracellular delivery of drugs and reduce their efficacy [12]. Various approaches, including nanoparticles, have been studied to overcome resistance to chemotherapeutic drugs [14].

## 2. Nanoparticles and Liposome-Mediated Drug Delivery

**Figure 1:**
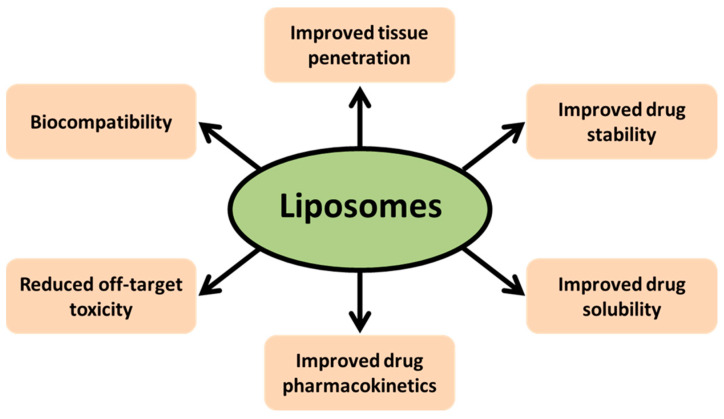
Advantages of liposomes as drug delivery systems.

The effectiveness of many chemotherapeutic drugs can be limited by their rapid metabolism, their toxic side effects, and the development of resistance [15]. To overcome these limitations, nanoparticles, such as liposomes, have been used to improve the therapeutic efficacy of various chemotherapeutic drugs. Liposomes provide several advantages, including improved pharmacokinetic properties of the encapsulated drug, long circulation time, and passive targeting and disposition in tumors and inflammatory sites due to the enhanced permeability and retention (EPR) mechanism. They can also reduce systemic toxicity associated with the free drug. In addition, liposomes can improve the solubility of drugs and provide slow and sustained release of encapsulated drugs (Figure 1). However, it is worthy to note that despite their efficacy in treating various cancers, nanoparticles, including liposomes, still have some potential toxicity and lack specific targeting and disposition [16,17,18].

**Figure 2:**
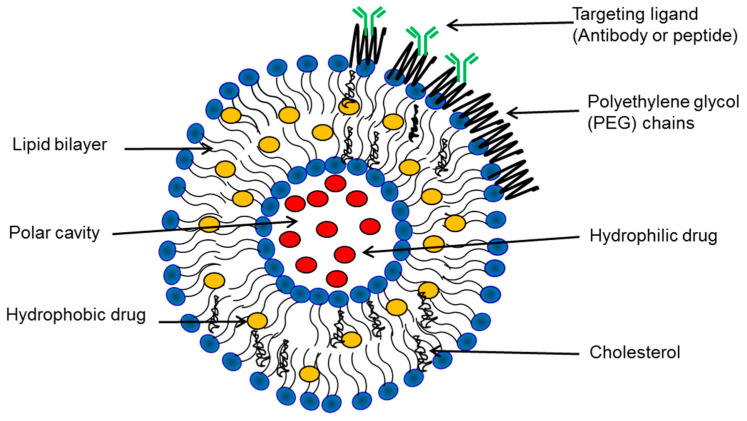
Schematic of liposomes and their different components.

Since their discovery in the 1960s by the late Alec Bangham, liposomes have been extensively studied as drug delivery systems, and they continue to be investigated in various research areas [19]. In the early 1970s, the work lead by Gregory Gregoriadis successfully delivered enzymes into the lysosomes of tissues in the reticuloendothelial system (RES) [20,21]. His following work, along with his colleagues, introduced the potential of exploiting liposomes as a drug delivery system [22,23,24]. Liposomes are considered to be one of the most successful drug-carrier systems, and several liposomal formulations are actively marketed or are in clinical trials [25]. Liposomes result from the self-assembly of phospholipids in an aqueous media, resulting in closed bilayered structures with an aqueous cavity and one or more bilayer phospholipid membranes (Figure 2) [26,27].

Phospholipids are the main components of cell membranes, which make them biocompatible. In addition, their amphiphilic properties enable self-assembly into bilayer membranes in aqueous environments [28,29]. These unique properties make phospholipids suitable for drug delivery systems such as liposomes. Phospholipids are characterized by their phase transition temperature (T_C_), which is the temperature at which phospholipids transit from gel crystalline to liquid crystalline states [30]. The T_C_ depends on many factors, such as the nature of the polar head group of phospholipids, the length of their aliphatic chains, and the presence of unsaturation in their hydrocarbon chains [31].

Liposomal formulations usually include cholesterol incorporated into the lipid bilayer to decrease membrane fluidity and control the rate of drug release. Cholesterol can reduce the rotational freedom of the phospholipid hydrocarbon chains, which limits liposome interactions with plasma proteins and subsequent loss of the encapsulated material [32,33,34,35]. Cholesterol also plays an essential function in regulating the biophysical states of the phospholipids in the liposomes by controlling the lipid organization and phase behavior. Cholesterol decreases the order of phospholipids in the crystalline gel phase and increases the order in the liquid crystalline phase [31,36]. Studies have shown that adding cholesterol to liposomal formulations shifts the T_C_ of phospholipids to a lower temperature, and a cholesterol composition above 30% abolishes the T_C_. Moreover, adding cholesterol increases the stability of liposomes and limits their leakage after systemic administration [36,37].

The unique structure of liposomes allows hydrophilic drugs to be retained in the aqueous interior. Hydrophobic drugs are usually inserted into the liposome bilayer; however, caution should be taken with this approach because high drug concentrations can disrupt liposomes (Figure 2). Amphipathic drugs can also be encapsulated in liposomes, provided the drug is partitioned between bilayer and aqueous phases [38].

Additional advantages of liposomes include biocompatibility, biodegradability, and decreased drug side effects [25]. Liposomes allow for controlled drug release and protection from rapid metabolism and clearance. Liposomes are also associated with improved patient compliance because of a decreased frequency of drug administration as compared to unencapsulated drugs [32,39,40]. Liposomes, like other nanoparticles, have some disadvantages, including possible carrier toxicity [41]. Typically, the toxicity of liposomes is lower, as compared to other nanoparticles. Liposomes are primarily composed of phospholipids, and other lipids, that are generally recognized as safe (GRAS), as well as biocompatible, biodegradable, and non-immunogenic [42,43]. Another limitation of liposomes is their preparation on a large industrial scale with reproducible properties [44]. The stability of liposomes constitutes another limitation, and lyophilizing the produced lipid vesicles is one of the proposed solutions to overcome this limitation [45,46].

## 3. Biophysical Characterization of Liposomes

In general, physical characterization of liposomes and nanoparticles (NPs) is necessary to accurately correlate a particular property of a nanoparticle with biological reactions. Particle size (or diameter) and surface area properties of NPs are key parameters for their characterization.

### 3.1. Transmission Electron Microscopy (TEM)

Electron microscopy techniques, such as transmission electron microscopy (TEM), can provide accurate size measurements of liposomes. However, this technique is unable to resolve any organic surface ligands conjugated to liposomes because of their low electron density. As such, the size determined by TEM is mainly of the core of liposomes. TEM is also limited in that it requires a high vacuum that may result in liposome aggregation during their preparation for imaging [47,48,49]. TEM suffers from limited sampling, and the data reported are not necessarily representative of all the NPs in one formulation [50]. Therefore, multiple methods should be used to determine the size of liposomes.

### 3.2. Dynamic Light Scattering (DLS)

Dynamic light scattering is one of the most commonly used methods to determine the size of liposomes. DLS can measure particle size, particle size distribution and surface charge, or zeta potential. DLS requires that the material be dispersed in a solvent and that all liposomes are individually dispersed. DLS is based on the fact that dispersed nanoparticles are in a continuous Brownian motion, and the illumination with a laser results in scattered light fluctuations at a rate dependent upon the size of the particles. The diameter measured by DLS is called the hydrodynamic diameter, which refers to how a particle diffuses within a fluid. While DLS is simple and quick, it has a limitation of low sensitivity towards small particles (i.e., lower limit for DLS measurement is around 10 nm) and interference from light-absorbing species [49].

### 3.3. Fluorescence Correlation Spectroscopy (FCS)

Fluorescence correlation spectroscopy is used to measure the hydrodynamic diameter of nanoparticles that are either intrinsically fluorescent, or labeled with fluorescent dyes [51]. FCS measures the duration of brief bursts of photons from individual nanoparticles passing through a small focal volume of typically 1 fL (10^−15^ L), and the size can be determined using autocorrelation analysis [52].

### 3.4. Encapsulation Efficiency (EE)

EE is defined as the ratio of the amount of encapsulated drug to the initial amount of drug added in the liposome formulation [46,53]. Once prepared, liposome formulations are usually a mixture of encapsulated and un-encapsulated drug fractions. In order to determine the EE of liposomes, a first step is to separate the unencapsulated drug fraction from the encapsulated drug. This can be achieved using size-exclusion chromatography [54]. This method separates the two fractions based on the differences in the size between the drug-encapsulated liposomes and the free drug, where the unencapsulated drug may be retained in the gel while loaded liposomes are not [55,56,57]. Loaded liposomes can also be separated from a free unencapsulated drug using a dialysis membrane with the appropriate cut-off pore size [58]. Once drug-loaded liposomes are separated from a free drug, the lipid bilayer is disrupted, and the released drug is then quantified using various techniques such as spectrophotometry and high-performance liquid chromatography (HPLC) [59].

### 3.5. Phospholipid Content

Phospholipid content of liposomes can be determined by spectrophotometric techniques using several methods, such as the Bartlett phosphate assay [60]. This method is based on the colorimetric measurement of inorganic phosphate. The phospholipid content of liposomes is measured after acid-based destruction of the phospholipid to inorganic phosphate. This step is followed by the addition of ammonium molybdate and the conversion of inorganic phosphate into phospho-molybdic acid. Ammonium molybdate is then reduced into a blue-colored complex that can be measured spectrophotometrically at 830 nm [60]. In vitro stability of liposome-encapsulated drugs can be assessed by dialyzing samples of liposomal formulations and quantifying the drug content at different time points, as indicated above [61].

Additional biophysical properties of liposomes that affect encapsulation of drugs in liposomes and their efficacy include phospholipid composition and lamellarity, bilayer rigidity, lipid-to-drug ratio, in vitro drug release, and drug-liposome interactions. A more detailed discussion of these properties is beyond the focus of this review. However, these properties were discussed in a couple of studies, and the reader is referred to these reviews that focus on these aspects of liposomes [62,63,64,65,66,67,68].

## 4. Preparation and Properties of Liposomes

Over the years, research publications reported the use of different methods to make liposomes. Most techniques used in liposome preparation include the dissolution of phospholipids in their appropriate organic solvents, followed by the removal of organic solvents to allow liposomes to form [69]. There are several different methods used to load drugs into liposomes, and these differ depending on the hydrophilicity or hydrophobicity of the drug being encapsulated, as well as whether liposomes are manufactured at a small laboratory scale or an industrial scale [70]. Hydrophobic drugs, such as paclitaxel and docetaxel, have been loaded into liposomes using the lipid film hydration method with sufficient encapsulation [71,72]. Hydrophilic drugs are encapsulated into liposomes using passive or remote (active) loading. Passive loading involves entrapping drugs as the lipid films are hydrated; however, a major limitation of this method is the low encapsulation efficiency, as most hydrophilic drugs remain entrapped in the external aqueous compartment [73,74]. Below are examples of commonly used methods in the preparation of liposomes.

### 4.1. Thin Lipid Film Hydration

Thin lipid film hydration is the simplest, oldest, and one of the most widely used methods at the research laboratory scale [75,76,77,78]. Phospholipids dissolved in organic solvents are subject to the removal of organic solvent via evaporation, resulting in a thin lipid film. Hydration of the lipid film results in heterogeneous liposomes dispersed in the aqueous solvent. Several techniques can reduce their heterogeneity and narrow their size distribution, including sonication and multiple extrusions through polycarbonate membranes [75,76,79,80]. This lipid film hydration method is easy, simple, and reproducible. However, one major limitation of this method is the low EE of hydrophilic drugs. Applying remote loading techniques can increase the EE, which involves changing the transmembrane pH or ionic gradients across the liposomal membranes. This method was successfully used in the encapsulation of anti-cancer drugs including doxorubicin [81,82].

### 4.2. Reverse Phase Evaporation

Reverse phase evaporation is a relatively simple method that is used to improve the EE of drugs into liposomes. This method is based on the formation of an emulsion of an aqueous phase (containing the drug) and an organic phase (containing the lipid), followed by the evaporation of the organic solvent, and the formation of an aqueous suspension containing the assembled liposomes [83,84]. Examples of drugs that have been encapsulated using this method include the anti-Alzheimer drug tacrine hydrochloride [85], the anti-cancer drug doxorubicin [86], and carboplatin [87].

### 4.3. Dehydration-Rehydration

Dehydration-rehydration is another method used to improve drug EE. This approach consists of dispersing preformed and lyophilized liposomes in an aqueous solvent [38]. This method consists of first making liposomal suspensions with small unilamellar vesicles followed by lyophilization. Upon rehydration, the liposomes reform and passively entrap the compound of interest. This method requires the addition of suitable lyoprotectants to preserve the liposomes’ bilayers integrity and prevent aggregation of liposomes during rehydration [88,89]. Radiocontrast agents (e.g., diatrizoate and iotrolan), DNA, and RNA have been encapsulated in liposomes with this method [90,91].

The methods described above have been used successfully in small-scale research laboratories. However, the large-scale industrial manufacturing of liposomes has not been as successful for many reasons. The industrial production of liposomes faces many limitations such as the batch-to-batch variability, which affects the chemical, physical, and performance qualities of the product. In addition, it is a long and laborious process that involves several operations. Additional levels of complexity are usually related to the additional testing performed prior to, during, and after the manufacturing, storage, and clinical applications as well as the lack of well controlled good manufacturing practices (GMPs) [70,92]. Recently, the preparation of drug-loaded liposomes has been revolutionized by the state-of-the-art microfluidic systems [93].

### 4.4. Microfluidic Techniques

Microfluidic techniques, especially microfluidic hydrodynamic focusing (MHF), have shown great potential to achieve high quality control over the physical properties of liposomes, including their uniformity in size and narrow size distribution. This technique is suitable for both hydrophilic and hydrophobic drugs and is based on solvent displacement methods. This technique uses a microfluidic device that introduces the lipid solution through a central channel, followed by the flow of aqueous solution. The aqueous solvent will then replace the organic solvent and promote the self-assembly of liposomes. This technique has the advantage of producing uniformly sized unilamellar liposomes because of the ability to change the flow rate of the lipid solution in the organic solvent and aqueous phase [92,94]. Other techniques to determine nanoparticle size includes atomic force microscopy (AFM), nanoparticle tracking analysis (NTA), absorption spectroscopy, and analytical ultracentrifugation [95,96,97,98].

## 5. Active Loading vs. Passive Loading of Drugs in Liposomes

Active or remote loading has been used to increase the EE of hydrophilic drugs. This method was first used by the Deamer research group, who used a pH gradient across the liposomal membranes to load catecholamine remotely into liposomes [99]. Remote loading is based on the fact that uncharged drugs will cross the liposomal membrane and become protonated and entrapped inside the polar cavity. This change in protonation means that these drugs can no longer easily diffuse across the bilayer membrane. Effective remote loading requires drugs that have a *p*K_a_ ≤ 11 and a distribution coefficient (logD) within −2.5 to 2.0 at pH 7 [38,100]. Successful remote loading was achieved with the anti-cancer anthracycline drug, doxorubicin, known as Doxil^®^—an FDA-approved liposomal formulation used for the treatment of AIDS-related Kaposi’s sarcoma, recurrent ovarian cancer, and metastatic breast cancer [101,102]. Doxorubicin was stably encapsulated in this liposomal formulation using an ammonium sulfate pH gradient across the liposomal membrane. Doxorubicin is a weak base in the outer compartment and exchanges with the ammonium ions from the inner compartment. Once inside the polar cavity, doxorubicin is precipitated as a sulfate salt, which extends the controlled and sustained release of the drug from the liposomes, and extends its half-life to more than 50 h in plasma [102]. In addition, Doxil^®^ decreased cardiomyopathy associated with free doxorubicin and allowed patients to receive fewer doses of the drug because of the extended half-life of the liposomes [103]. Table 1 lists several drugs that have been encapsulated in liposomes and approved by the U.S. Food and Drug Administration (FDA) and European Medicines Agency (EMA) for the treatment of different diseases or are currently in clinical trials (Table 2).

The surface charge and liposome composition play an important role in the fate of liposomes. Anionic and neutral liposomes have been shown to escape renal clearance; however, cationic liposomes can have specific interactions with various anionic species in the blood that enhance their clearance through the reticuloendothelial system (RES). Neutral liposomes have been shown to accumulate in solid tumors [141]. Increasing the mol % of cationic lipids in liposomes can induce aggregation through electrostatic interactions between the liposomes and the anionic species in the circulation, resulting in reduced liposome disposition at the target site [142]. Studies have also shown that cationic liposomes preferentially accumulate in the angiogenic tumor vessels, and they may be efficient drug carrier systems to target blood vessels of solid tumors. Because of their extravasation, neutral and anionic liposomes have been suggested as potential drug carriers to the extravascular compartments of tumors [143].

## 6. Strategies for Targeting Liposomes to Tumors

Liposomes have emerged as efficient carrier systems for therapeutic agents, owing, in part, to some of the unique properties discussed above. Various strategies have been developed to target liposomes to tumor sites. Some of these strategies involve using passive targeting and active targeting via surface functionalization as well as using various stimuli to trigger drug release from liposomes.

### 6.1. Liposomes and the EPR Effect (Passive Targeting)

**Figure 3:**
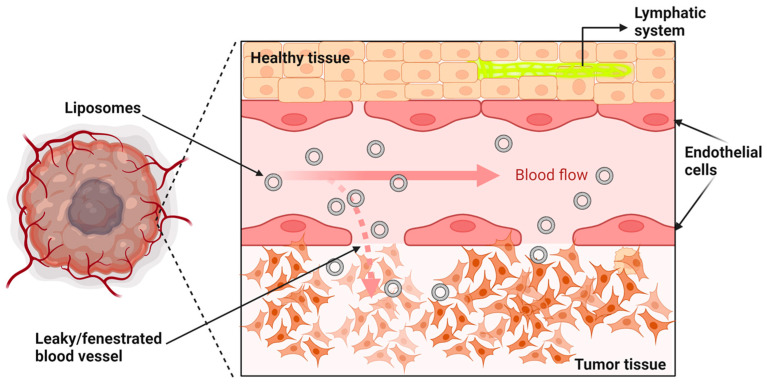
Tumor vasculature leakage. Tumor vasculature exhibits structural abnormalities, fenestrated blood vessels, and absence of a lymphatic system (**bottom**) compared to normal/healthy vasculature (**top**). Image was created with BioRender.com (accessed on 1 February 2023).

Nanoparticles, such as long circulating liposomes, take advantage of the leaky nature of the blood vessels in tumor tissues. Because tumor blood vessels have increased fenestrations, liposomes can passively cross the capillary endothelial barrier and reach the interstitial space [144,145]. In normal non-tumor tissues, vascular endothelial cells are tightly connected and have small para-cellular gaps in the 5–10 nm range. In contrast, larger gaps exist between endothelial cells in tumor blood vessels, ranging from 100 to 700 nm, depending on the cancer [146,147]. In addition, because of their disorganized vascular architecture, solid tumors lack a functional lymphatic system (Figure 3). The combination of the leaky tumor vasculature and the limited lymphatic drainage is called the enhanced permeability and retention (EPR) effect, which allows the passive disposition and accumulation of liposomes into the tumor site [145,148,149,150,151,152,153].

First-generation, or conventional, liposomes had limited tumor disposition because of their rapid clearance by the reticular endothelial system (RES), and their opsonization by plasma proteins. In addition, these liposomes suffered from drug leakage during their systemic circulation [154]. Over the years, changes were made to improve these liposomes, including composition and surface modification, to produce the second-generation liposomes. These liposomes had improved stability, disposition, and efficacy compared to first generation liposomes. Cholesterol was added in the lipid bilayers of liposomes to increase their rigidity and reduce drug leakage [25,155]. The incorporation of polyethylene glycol or PEG (PEGylation) provided a steric protection of liposomes from electrostatic and hydrophobic interactions with plasma proteins, which decreased uptake by RES. In addition, PEGylation extended the circulation time of liposomes, allowing for a more effective drug delivery in vivo. These long-circulating liposomes were therefore named “stealth liposomes” [27,39,156,157].

The first stealth liposomal formulation to be approved for cancer therapy in the United States (1995) and European Union (1996) was Doxil^®^/Caelyx^®^ [102,113]. Doxil^®^ offers reduced cardiotoxicity and myelotoxicity in comparison to free doxorubicin, while achieving higher drug concentrations in tumors by using a liposomal composition of HSPC:CL:MPEG 2000-DSPE (calc. molar ratio 3:2:0.9, *w*/*w* 3:1:1) [112,158]. While the Doxil^®^/Caelyx^®^ liposomal formulation is clinically efficacious, efforts have been made to change the formulation in order to improve the pharmacokinetic properties. Lipo-Dox^®^ was created with a similar lipid molar ratio to Doxil^®^/Caelyx^®^, except the formulation consists of DSPC instead of HSPC (HSPC:CL:MPEG-DPSC, 3:2:0.3) [114,115]. DSPC has a higher phase transition temperature than HSPC [159], and DSPC consists of one type of long-chain fatty acid (i.e., stearic acid, C18). On the other hand, HSPC has a varied fatty acid composition of palmitic (C16) and stearic acid (C18) [160,161]. In a phase I study, replacing HSPC with DSPC in the liposomal doxorubicin formulation increased the half-life, as well as reduced the volume of distribution and clearance rate, which overall offered a better plasma area under the curve (AUC) performance than the reported plasma AUC for Doxil^®^/Caelyx^®^ [162,163]. While stomatitis was noted as a new dose-limiting toxicity at 50 mg/m^2^ with the Lipo-Dox^®^ formulation, which is not an observed limitation with Doxil^®^/Caelyx^®^, combinations of Lipo-Dox^®^ with other cancer treatments have been efficacious for patients with ovarian, AIDS-related Kaposi’s sarcoma, and breast cancer in Taiwan [164,165,166]. Zolsketil^®^, a recently authorized bioequivalent to Caelyx^®^, is available in the European Union to treat advanced ovarian cancer, breast cancer, multiple myeloma, and AIDS-related Kaposi’s sarcoma [128]. Comparing the drug leaflets, Zolsketil^®^ uses the same excipients as Caelyx^®^/Doxil^®^ (i.e., HSPC, MPEG 2000-DSPE, CL, ammonium sulphate, histidine, sucrose, water, HCl, NaOH), but it is unclear at this time the excipient molar ratio or other modifications to the preparation of the liposomes that would distinguish the two stealth liposome formulations.

Although proven to be clinically useful, stealth liposomes depend mostly on their passive accumulation into tumor tissues; they lack the ability to control cellular uptake and drug release and rely only on passive drug efflux, which may result in limited efficacy [25]. For example, SPI-77 is a stealth liposome of cisplatin with a similar formulation to Doxil^®^ (HSPC:CL:MPEG2000-DSPE), but it has not progressed beyond phase II clinical trials. While SPI-77 demonstrated a better toxicity profile over the conventional toxicities observed with cisplatin alone in non-small cell lung cancer patients, most patients in the phase II clinical studies did not respond to SPI-77 [167,168]. The third, or “new generation”, liposomes use ligand-mediated targeting or active targeting to improve biodistribution and liposome-mediated drug delivery at tumor sites [169].

### 6.2. Active Targeting of Liposomes

Most nanomedicines are using passive mechanisms, such as the EPR effect, to target tumors. Many of these have failed to get FDA approval for clinical use. Passive targeting of liposomes relies only on the pathophysiological properties at the tumor site and has limitations that include decreased efficacy and/or off-target toxicity [170]. One reason for this lack of clinical efficacy is that passively targeted liposomes lack true specificity for the tumor cells themselves. This has led several researchers to focus on more precise forms of targeting liposomes, such as active targeting. Active targeting uses molecular approaches to directly target tumor cells via interactions with tumor-specific markers [171,172]. Actively targeted liposomes are usually prepared by conjugating targeting moieties such as monoclonal antibodies, fragments of antibodies, or peptides to their surface [146]. This approach is a promising strategy for cancer therapy [146,173,174,175]. Active targeting utilizes specific pathological changes in the tumor microenvironment such as the overexpression of several proteins. Therefore, liposomes targeting these markers can be selectively taken up by cells that overexpress these proteins to achieve improved drug delivery [176,177,178,179]. While active targeting has the ability to target cells once liposomes are in the tumor microenvironment, it actually has no tumor targeting ability. Liposomes still rely on their passive movement to reach tumors. Recently, transcytosable nanomedicine has emerged as an alternative approach that has the potential to cross the vascular wall and diffuse more efficiently within the tumor tissue. The design of transcytosable nanomedicines depends on various forms of transcytosis, including receptor-mediated, adsorption-mediated, or fluid-mediated transcytosis [180,181].

Targeted drug delivery to cancer cells has gained significant interest and shown great potential due to the various overexpressed target proteins on cancer cells. Examples of these targets include human epidermal growth factor receptor 2 (HER2), epidermal growth factor receptor (EGFR), transferrin receptors, epithelial cell adhesion molecule (EpCAM), and vascular receptors [179,182]. The conjugation of antibodies to the surface of liposomes creates immunoliposomes that show enhanced cell binding and internalization compared to untargeted liposomes [174]. The majority of immunoliposomes currently studied are used for the delivery of anticancer drugs [183]. Antibodies are usually conjugated to PEG, and not to the liposomal phospholipids, to overcome the steric hindrance possibly caused by PEG interference with antibody-target protein interactions. Thus, the ligand is extended outside the PEG layer and is more accessible for binding to its target [183].

The conjugation of monoclonal antibodies (mAbs), or their Fab fragments against HER2, improved drug delivery to tumor sites and showed therapeutic efficacy in various HER2-overexpressing mouse xenograft models [184,185]. Doxorubicin-loaded liposomes conjugated to the nucleosome mAbs (2C5) showed increased antitumor efficacy in both in vitro and in vivo models of various over-expressing nucleosome tumors compared to non-actively targeted liposomes [186,187,188]. PEGylated liposomes conjugated to the internalizing receptor CD19, which is overexpressed in various B-lymphoid cancers, showed improved efficacy in a human CD19+ B-lymphoma mouse model compared to non-antibody conjugated liposomes [189,190].

### 6.3. Local Stimuli to Trigger Drug Release from Liposomes

Strategies also exist to increase drug release from liposomes after they accumulate in the tumor. Many of these strategies take advantage of pathological changes in the tumor microenvironment, such as altered pH, increased temperature, and overexpression of proteolytic enzymes such as secretory phospholipases [176,177]. External stimuli can also be applied to enhance or trigger drug release from liposomes [146]. pH-sensitive co-polymers can be added in liposomal formulations that are stable at a physiological pH, but these will be hydrolyzed at an acidic pH of 6 and lower, which is commonly found in the tumor microenvironment. Examples of polymers used in pH-responsive liposomes include poly (acrylic acid) and poly (methacrylic acid) [146,191]. Temperature-triggered drug delivery from liposomes involves local heating of the tumor site to increase tumor permeability by increasing vascular pore size. This increases liposomal extravasation and accumulation in the tumor microenvironment [192,193]. Temperature-sensitive liposomes are usually prepared by incorporating thermosensitive lipids with a specific gel-to-liquid phase transition temperature, such as dipalmitoylphosphatidylcholine (DPPC). These thermosensitive liposomes have been shown to release more than 80% of encapsulated methotrexate in the tumor site after raising the temperature from 37 °C to 41 °C [146,194,195]. ThermoDox^®^ (Celsion, NJ, USA), another thermosensitive liposomal formulation, showed improved doxorubicin delivery and efficacy in mouse models and has advanced to phase III clinical trials for treating hepatocellular carcinoma and breast cancer [194,196].

Taking advantage of specific pathological changes in the tumor microenvironment can increase drug release from liposomes, such as the overexpression of enzymes (e.g., matrix metalloproteinases (MMPs) and phospholipase A_2_) [78,197]. The activity of these enzymes can mediate the uptake and release of encapsulated drugs from enzyme-sensitive or responsive liposomes [198]. Despite the extensive research and the development of different liposome formulations, the sub-optimal potency is still a major limitation of liposomes. For instance, the most successful nanomedicine, Doxil^®^, can only achieve modest benefits. Additional work is needed and has to focus on how to improve the therapeutic efficacy of liposomes. While these strategies exist to increase drug delivery, another factor that is limiting drug release includes the PEG layer in stealth liposomes.

## 7. PEGylation of Liposomes

PEGylation offers stealth properties to liposomes, including evasion of the mononuclear phagocytic system and extended circulation times that are responsive to PEG length and density [199]. Increasing the percent of grafted PEG on liposomes (i.e., 2–5 mol%), and using PEG_2000_ or PEG_5000_, markedly reduces protein adsorption, phagocytosis, and cellular adhesion of erythrocytes, lymphocytes, and macrophages [200,201]. However, some of the beneficial properties of PEGylated liposomes can create a few challenges for maximizing drug delivery, cell uptake, and endosomal escape.

### 7.1. Accelerated Blood Clearance

In animal models, increased blood clearance and increased accumulation in the liver and spleen can occur after a second injection (i.e., <4 weeks from the 1st injection) of PEGylated liposomes, which is known as the accelerated blood clearance phenomenon [202,203,204]. In addition, the second injection (i.e., <1 week) of PEGylated liposomes results in significantly increased IgM production in rats [205], despite using different types of lipids (i.e., EPC, SPC, ESM, HSPC, DPPC) [204]. Interestingly, the accelerated blood clearance phenomenon is not associated with Doxil^®^ in clinical practice, but the significance of this phenomenon in clinical studies is still an area of debate [206,207]. Moreover, various strategies are still being pursued, such as employing alternative PEG-lipid/cholesterol derivatives (e.g., PEG-cholesteryl hemisuccinate) or a cleavable PEG (e.g., pH), to significantly reduce the accelerated blood clearance phenomenon [208,209,210]. One of the additional benefits of using a cleavable PEG coating is that it can help improve cargo delivery to target cells.

### 7.2. Cell Uptake and Cargo Delivery of PEGylated Nanoparticles

PEGylation of nanoparticles can introduce varying degrees of inhibitory effects on cell uptake, tumor-targeted efficiency, and endosomal escape; however, not all PEGylated formulations lead to these effects, and it can vary depending on the target cells [211,212,213,214]. The inhibitory effects of PEGylation have been reported with solid nanoparticles [215,216,217], lipid-DNA complexes [212,218,219], and chemotherapeutic loaded liposomal formulations [220,221], and recent reviews are available to provide greater depth in this topic [222,223].

PEG length and density play an essential role in cell uptake and endosomal escape. Starting simply on the level of measuring how PEGylation impacts liposome-to-liposome fusion, Holland et al. performed in vitro fusion assays measuring the changes in resonance energy transfer from mixing fluorescently labeled liposomes (i.e., Rh-PE and NBD-PE) with non-fluorescent liposomes, followed by the addition of CaCl_2_ to promote liposome fusion [224]. They observed that increasing the mol % of the PEG-lipid incorporated in the liposomes reduces the maximal % vesicle fusion, and 2 mol % was enough to completely inhibit vesicle fusion. Additionally, the rate of fusion decreased with increasing acyl chain length and saturation in the PEG-lipid conjugate. Increasing the PEG length (i.e., 2000 to 5000) markedly reduced the maximal % fusion achieved with increasing the mol % of the PEG-lipid conjugate. Interestingly, Brandenberger et al. observed that PEG_5000_ coated gold nanoparticles had significantly less cell uptake in human lung carcinoma cells (A549) in comparison to plain gold nanoparticles [215]. Furthermore, Song et al. reported that increasing the PEG length (i.e., 220 to 3400) or increasing the hydrophobic anchor of the conjugated PEG lipid (i.e., ceramide C8 to C20) in unilamellar DNA/lipid complexes markedly inhibited gene transfection in liver (HepG2) and cervical cancer (HeLa) cells [212]. Monitoring cell uptake of fluorescently labeled lipid and DNA complexes revealed that while the lipid/DNA complexes were endocytosed, the longer PEG and ceramide chain lengths inhibited cargo release, which resulted in complexes unable to escape the perinuclear region. Hence, the PEG density and length need to be balanced to support optimal cargo delivery.

In the area of liposome-based chemotherapeutics, PEGylation has offered reduced accumulation in the liver, spleen, and heart over free drug, but some studies report that tumor-targeting efficiency (T_e_ = AUC_tumor_/AUC_plasma_) is reduced in comparison to non-PEGylated liposomal formulations. For example, Parr et al. reported that DSPC/CL doxorubicbin liposomal formulations, with or without PEG_2000_-PE, achieved similar drug accumulation levels by day 4 in BDF-1 mice bearing Lewis Lung carcinoma, but the formulation without PEG_2000_-PE achieved significantly higher drug accumulation levels in tumors earlier (1 h to 48 h) in the animal study [221]. Therapeutic efficacy was not significantly different between formulations with or without PEG_2000_-PE. The tumor targeting efficiency (T_e_) of the DSPC/CL doxorubicin formulation without PEG was nearly 2-fold greater than the T_e_ value (0.76) of the DSPC/CL/PEG_2000_-PE formulation (0.40). Hong et al. also noticed a similar observation of lower tumor targeting efficiency with their own comparison studies of PEGylated and non-PEGylated liposomal doxorubicin in C-26 carcinoma tumor-bearing mice [225]. By 24 h, the doxorubicin concentration in the tumor was higher than plasma with the non-PEGylated formulation, while the drug concentration remained lower than plasma with the PEGylated formulation. Overall, the doxorubicin liposomal formulation without PEG offered a greater accumulation of drug in tumors over 72 h than with PEG. The drug targeting efficiency (T_e_) was greater than 2-fold for non-PEGylated liposomal doxorubicin (0.87) in comparison to the PEGylated liposomal doxorubicin (0.31). Moreover, while both liposomal formulations were significantly more efficacious at extending survival of C-26 mice in comparison to free drug, there was no significant difference in survival with or without PEG in the liposomal formulation. Of note, PEGylated liposomal doxorubicin had the greatest advantage over the non-PEGylated formulation with reducing drug accumulation in the liver and spleen.

### 7.3. Cleavable PEG Coatings

In an effort to still use the steric stabilization offered by PEGylation, others have taken the approach to create cleavable PEG-lipid linkages in order to shed the PEG coating. One effective strategy to overcome these limitations is by installing acid-labile acetal, hydrazine, hydrazone, or vinyl ether linkages [226,227] between PEG and the lipid or polymer, which takes advantage of the slightly acidic tumor microenvironment (pH 5.6–7 [228,229]) as well as endosomes (pH 5.5–6.5 [230]). For example, Kanamala et al. linked PEG_2000_ to 1,2-dipalmitoyl-sn-glycero-3-phosphoethanolamine (DPPE) via an acid-labile hydrazine bond, which was incorporated at 5 mol% into DOPE, DSPC, CHEMS, cholesterol liposomes (4:2:2:2 molar ratio) with calcein loaded in the core, and Nile Red in the bilayer [231]. Visible by live cell imaging and confocal fluorescence microscopy, the non-cleavable and cleavable PEG pH-sensitive liposomes (pSLs) were both internalized by pancreatic cancer cells (MIA PaCa-2) after 30 min, but calcein release was clearly seen in the cytoplasm by 1 h with the cleavable PEG liposomes. On the other hand, the liposomes with covalently linked PEG remained as a punctate distribution. Using the same formulation, gemcitabine was encapsulated in the cleavable PEG pSLs and demonstrated increased cytotoxicity over the covalently linked PEG pSLs in MIA PaCa-2, and even performed better than free gemcitabine in a glioblastoma cell line (U-87) after 24 h of exposure. An in vivo biodistribution study with CD-1 nude mice bearing MIA PaCa-2 tumors demonstrated a statistically significant increase in gemcitabine accumulation in the tumor with cleavable PEG pSLs than the covalently linked PEG pSLs and free drug. While gemcitabine levels were significantly increased in the liver with both types of pSL formulations in comparison to the free drug, both formulations offered significantly less drug accumulation in the spleen and heart at 4 h. In addition to enhancing drug accumulation, endosomal escape, and cell uptake, a cleavable PEG strategy can be useful to shield targeting moieties for enhancing cell uptake upon reaching the tumor site.

Strategies to target liposomes (e.g., peptide-targeted liposomes) or use fusogenic materials to increase tumor cell uptake and drug delivery can be concealed with a cleavable PEG polymeric layer that would be shed upon an external stimulus (i.e., lower pH or reducing conditions) [232,233,234,235] and expose the targeting ligands to guide the liposomes to the tumor cells. Hak et al. noticed that keeping the PEG surface density to <10 mol% in 100 nm of α_v_β_3_-integrin targeted (i.e., using an RGD peptide) nanoemulsions was optimal for targeting tumor sites [236]. Consistent with the idea that reducing the PEG density or shedding PEG supports targeted delivery, Geng et al. recently reported the in vitro and in vivo performance of an α_v_β_3_-integrin targeted (i.e., RGD peptide), PEG cleavable, doxorubicin liposomal formulation that uses near-infrared (NIR) light to shed the PEG thermo-labile linker, 4,4′-azobis (4-cyanovaleric acid). Upon exposure to NIR, the thermo-labile, PEGylated doxorubicin liposomes demonstrate significantly increased cell uptake, enhanced drug accumulation at the tumor site, and efficacy in H22 tumor-bearing mice when compared against doxorubicin or the thermo-stabile PEGylated doxorubicin formulation [237].

Yang et al. designed a charge reversed doxorubicin liposomal formulation (CRDOXIL) using a 4:1:1 weight ratio of HSPC, Chol, and cleavable PEG_2000_ [238]. PEG_2000_ is attached to a distearoyl via a diakylmaleamidic amide linkage, which falls apart at a low pH to expose a primary amine. Hence, these liposomes will primarily bear a positively charged surface upon encountering a low pH environment (pH 6.5), which is anticipated to facilitate cell uptake as well as endosomal escape. Indeed, the CRDOXIL formulation had increased cell uptake than the DOXIL (weight ratio of 4:1:1 with HSPC, CHOL, and DSPE-mPEG_2000_) based on fluorescence microscopy and flow cytometry. Moreover, while both DOXIL and CRDOXIL performed similarly with normal lung (BEAS-2B) and liver (L02) cell lines at pH 7.4, only CRDOXIL could demonstrate nearly the same reduction in cell viability to free doxorubicin when cultured with lung (A549) and liver (HepG2) cancer cells at pH 6.5.

The challenge in employing some of the common acid-labile chemical linkages (i.e., acid-labile acetal, hydrazine, hydrazone, or vinyl ether) is minimizing premature release of drug payloads at physiological pH, while ensuring the release is at pH 4.5–6.5 [223,239]. Unfortunately, many of the acid-labile linkages lack the stability to withstand the extended circulation times of stealth liposomes (e.g., 45 h in humans) at physiological pH [240]. A chemical linkage such as the PhosAm technology, which is stable for >70 h at pH 7.4 but rapidly hydrolyzes (t½ < 1 h) at pH 5.5, would be well matched with the hours of circulation that liposomes achieve [241,242]. Overall, there is promising potential in achieving a balance between the stealth properties of PEGylated liposomes and maximizing drug delivery by shedding the PEG layer.

## 8. Summary and Conclusions

Liposomes represent an attractive delivery system due to their physicochemical properties that allow overcoming various challenges and limitations with drug delivery. The use of liposomes to improve drug delivery has greatly impacted various biomedical areas. Liposomes have been shown to improve stability and biodistribution of therapeutic agents, overcome limitations to tissue and cellular uptake in target sites in vivo, and reduce systemic toxicity associated with non-encapsulated agents. However, despite the considerable preclinical work on liposomes, their translation into the clinic has progressed only incrementally. Future research will need to focus on addressing such translational limitations. This will require continuous communications and collaborations between experts in all stages of pharmaceutical development, including pre-clinical and clinical applications as well as toxicological evaluations.

## Figures and Tables

**Table 1:** Approval and Marketing History of Liposomal Drugs.

Product Name	Drug	Lipid Composition (Molar Ratio)	Indication	Approval Date	Marketing Status	Ref.
DaunoXome^®^ (US)	Daunorubicin	DSPC:CL (2:1)	AIDS-related Kaposi’s sarcoma	1995–1997 (EMA) 1996 (FDA)	US discontinued (2016), global on-demand access (2016, EU, UK, AU, NZ, HK)	[104,105,106,107,108]
DepoCyt^®^ (US, EU)	Cytarabine	CL:TR:DOPC:DPPG (*w*/*w*/4.4:1.2:5.7:1)	Lymphomatous meningitis	1999 (FDA) 2001 (EMA)	Withdrawn production by company (2017)	[109,110,111]
Doxil^®^ (US)/ Caelyx^®^ (EU) ^a^	Doxorubicin	HSPC:CL:MPEG 2000-DSPE (calc. 3:2:0.9, *w*/*w* 3:1:1)	AIDS-related Kaposi’s sarcoma, recurrent ovarian cancer, multiple myeloma, metastatic breast cancer (EU only)	1995 (FDA) 1996 (EMA)	Active (US, EU)	[102,112,113]
Lipo-Dox^®^ (TW)	Doxorubicin	DSPC:CL:MPEG 2000-DSPE (3:2:0.3)	AIDS-related Kaposi’s sarcoma, ovarian cancer, breast cancer, multiple myeloma	1998 (TW)	Active (TW)	[114,115]
Marqibo^®^ (US)	Vincristine	SM:CL (60:40)	Acute lymphoblastic leukemia	2012 (FDA)	US discontinued (2020)	[104,116]
Mepact^®^ (EU)	Mifamurtide	DOPS:POPC (3:7)	Osteosarcoma	2009 (EMA)	Active (EU)	[117,118,119]
Myocet^®^ (EU)	Doxorubicin	PC:CL (55:45)	Metastatic breast cancer	2000 (EMA)	Active (EU)	[120,121,122]
Onivyde^®^/Nal-IRI (EU, US)	Irinotecan	DSPC:CL:MPEG 2000-DSPE (3:2:0.015)	Pancreatic cancer	1996 (FDA) 2016 (EMA)	Active (US, EU)	[123,124,125]
Vyxeos^®^/CPX-351 (EU, US)	Cytarabine: daunorubicin (5:1 mol. ratio)	DSPG:DSPC:CL (7:2:1)	Newly diagnosed therapy–related acute myeloid leukemia, acute myeloid leukemia with myelodysplasia-related changes	2017 (FDA) 2018 (EMA)	Active (US, EU)	[126,127]
Zolsketil^®^ (EU) ^a^	Doxorubicin	HSPC:CL:MPEG 2000-DSPE	Metastatic breast cancer, advanced ovarian cancer, multiple myeloma, AIDS-related Kaposi’s sarcoma	2022 (EMA)	Active (EU)	[128]

Notes: Molar ratios are based on the literature unless indicated as calculated (Calc.) from a drug label. AU: Australia; Calc.: calculated; CL: cholesterol; DOPC: dioleoylphosphatidylcholine; DOPS: dioleoyl-phosphatidylserine; DPPG: dipalmitoylphosphatidylglycerol; DSPC: distearoylphosphatidylcholine; DSPG: distearoylphosphatidylglycerol; EU: Europe; EMA: European Medicines Agency; HK: Hong Kong; HSPC: fully hydrogenated soy phosphatidylcholine; NZ: New Zealand; MPEG2000-DSPE: N-(carbonylmethoxypolyethylene glycol-2000)-1,2-distearoyl-sn-glycero-3-phosphoethanolamine sodium salt; TR: triolein sodium salt; SM: sphingomyelin; sPLA_2_: secretory phospholipase A2; POPC: 1-palmitoyl-2-oleoyl-phosphatidylcholine; PC: phosphatidylcholine; *w*/*w*: weight for weight. ^a^: bioequivalent. However, it is unclear if Lipo-Dox^®^ is currently used for treating myelomas.

**Table 2:** Liposomal Drugs in Clinical Trials *.

Product Name	Drug	Lipid Composition (Molar Ratio)	Conditions	Delivery Mechanism	Status	Ref.
L-NDDP/ Aroplatin™	*cis*-*bis*-neodecanoato-*trans*-R,R-1,2- diaminocyclohexane platinum (II)	DMPC:DMPG	B-cell lymphoma, malignant mesothelioma, pancreatic cancer, colorectal cancer, solid tumors	EPR	Phase I/II	[129]
BP1002 (US)	Antisense oligonucleotide against BCl-2	DOPC:ASO (20:1)	Acute myeloid leukemia, advanced lymphoid malignancies	EPR	Phase I	[130]
EndoTAG^®^ (EU, US, TW, UA)	Paclitaxel	DOTAP:DOPC (53:47)	Breast cancer, pancreatic cancer, liver cancer	Electrostatic	Phase II/III	[131,132]
PLM60 (US, CN)	Mitoxantrone	HSPC:CL:MPEG 2000-DSPE (*w*/*w* 3:1:1)	Advanced hepatocellular carcinoma, small-cell lung cancer, non-Hodgkin’s lymphoma, recurrent/refractory lymphomas	EPR	Phase I/II	[133,134]
ThermoDox^®^ (US)	Doxorubicin	DPPC:MSPC:MPEG2000-DSPE (90:10:4)	Hepatocellular carcinoma, colorectal cancer, pediatric cancer, liver neoplasms, pancreatic cancer, breast cancer	Temperature	Phase I/II/III	[135,136]
LiPlaCis (DK)	Cisplatin	DSPC:DSPG:MPEG 2000-DSPE (mol.% 70:25:5)	Adv./refractory solid tumors, metastatic breast cancer, prostate cancer, skin cancer	sPLA_2_ targeted	Phase I/II	[137]
Lipoplatin™	Cisplatin	SPC-3: DPPG: CL: MPEG2000-DSPE	Malignant pleural effusions	Fusogenic	Phase I	[138]
SPI-077	Cisplatin	HSPC:CL:MPEG2000-DSPE (51:44:5)	Ovarian cancer	EPR	Phase II	[139,140]

* Searched trials are listed as either “active”, “unknown”, “not yet recruiting”, “terminated”, or “completed” in the clinicaltrials.gov database as of 26 March 2023. The listed liposomal drugs under clinical evaluation are not exhaustive. ASO: antisense oligonucleotide; CL: cholesterol; DMPC: 1,2-dimyristoylphosphatidylcholine; DMPG: 1,2-dimyristoylphosphatidylglycerol; DOTAP: dioleoyl-3-trimethylammonium propane; DPPC: dipalmitoylphosphatidylcholine; DPPG: dipalmitoylphosphatidylglycerol; HSPC, hydrogenated soy phosphatidylcholine; MPEG2000-DSPE: α-(2-(1,2-distearoyl-sn-glycero(3)phosphooxy)ethylcarbamoyl)-ω-methoxypoly(oxyethylen)-40; MSPC: monostearoylphosphatidylcholine; SPC-3, soy phosphatidyl choline.

## Data Availability

Data is contained within the article.
